# Genome reannotation of Escherichia coli CFT073 with new insights into virulence

**DOI:** 10.1186/1471-2164-10-552

**Published:** 2009-11-22

**Authors:** Chengwei Luo, Gang-Qing Hu, Huaiqiu Zhu

**Affiliations:** 1 State Key Laboratory for Turbulence and Complex Systems, and Department of Biomedical Engineering, College of Engineering, Peking University, Beijing 100871, China; 2 Center for Theoretical Biology, Peking University, Beijing 100871, China; 3 Center for Protein Science, Peking University, Beijing 100871, China

## Abstract

**Background:**

As one of human pathogens, the genome of Uropathogenic *Escherichia coli *strain CFT073 was sequenced and published in 2002, which was significant in pathogenetic bacterial genomics research. However, the current RefSeq annotation of this pathogen is now outdated to some degree, due to missing or misannotation of some essential genes associated with its virulence. We carried out a systematic reannotation by combining automated annotation tools with manual efforts to provide a comprehensive understanding of virulence for the CFT073 genome.

**Results:**

The reannotation excluded 608 coding sequences from the RefSeq annotation. Meanwhile, a total of 299 coding sequences were newly added, about one third of them are found in genomic island (GI) regions while more than one fifth of them are located in virulence related regions pathogenicity islands (PAIs). Furthermore, there are totally 341 genes were relocated with their translational initiation sites (TISs), which resulted in a high quality of gene start annotation. In addition, 94 pseudogenes annotated in RefSeq were thoroughly inspected and updated. The number of miscellaneous genes (sRNAs) has been updated from 6 in RefSeq to 46 in the reannotation. Based on the adjustment in the reannotation, subsequent analysis were conducted by both general and case studies on new virulence factors or new virulence-associated genes that are crucial during the urinary tract infections (UTIs) process, including invasion, colonization, nutrition uptaking and population density control. Furthermore, miscellaneous RNAs collected in the reannotation are believed to contribute to the virulence of strain CFT073. The reannotation including the nucleotide data, the original RefSeq annotation, and all reannotated results is freely available *via *http://mech.ctb.pku.edu.cn/CFT073/.

**Conclusion:**

As a result, the reannotation presents a more comprehensive picture of mechanisms of uropathogenicity of UPEC strain CFT073. The new genes change the view of its uropathogenicity in many respects, particularly by new genes in GI regions and new virulence-associated factors. The reannotation thus functions as an important source by providing new information about genomic structure and organization, and gene function. Moreover, we expect that the detailed analysis will facilitate the studies for exploration of novel virulence mechanisms and help guide experimental design.

## Background

Uropathogenic *Escherichia coli *(UPEC) strains lead to 70-90% of the estimated annual 150 million community-acquired urinary tract infections (UTIs) [1]. As a member of UPEC, the complete genome of strain CFT073 (serotype O6:K2:H1) was sequenced in 2002 [GenBank: AE014075.1] [2], which has a 5,231,428 bp chromosome without plasmid and is 590,209 bp longer than the well-studied K-12 MG1655 strain. The difference in the CFT073 genome is mostly caused by five unique cryptic inserted prophage genomes that contain a large portion of virulence or virulence-associated genes, referred to as pathogenicity islands (PAIs) [3]. At the time of this writing, the release in RefSeq annotates 5,339 protein-coding genes, 89 tRNA genes, 21 rRNA genes, and 6 miscellaneous RNA genes [2]. In-depth analysis reveals that 3,190 genes (3,925,047 bp, 75.0%) are considered as conserved backbone genes, while the rest (1,306,391 bp, 25.0%), known as CFT073-specific islands, inserts into the backbone regions in an extensive mosaic manner. Regarding virulence and virulence-associated genes, the annotation includes 12 types of fimbriae, 7 autotransporters, and toxin operons such as *hlyCABD *and *upxBDA *[2]. Since its first release, the annotation not only presents an overview of the complexity of the pathogen's lifestyle, but also has served as a guide for experimental design.

However, several lines of evidence suggest a need for the reannotation of the *Escherichia coli *CFT073 genome, partially due to discoveries and corrections overtime for the original RefSeq annotation even updated with some minor corrections. For example, new autotransporter encoding genes and some vital population density control factors are missing from the annotation [4,5], while more and more novel small RNAs (sRNAs) that have recently been found to add to the complexity of virulence regulatory networks [6]. In addition, a computational estimation suggests that the annotation quality of the translation initiation site is surprisingly lower in this strain than in its close relative, K-12 MG1655 [7]. Moreover, similar observation, along with low annotation quality in CDSs, has been demonstrated in other *E. coli *strains (for example, APEC O1), by syntactic annotation methods [8]. Such an observation indicates that the highly diverse adaptive paths in different *E. coli *are responsible for the requirement of more sophisticated annotation methods rather than traditional ones. As a systematic issue, research on how CFT073 establishes its virulence during the UTI process needs a more comprehensive and precise picture of the genomic structure of this pathogen instead of piecemeal information. Therefore, a thorough reannotation of CFT073 is justified for future studies.

Reannotation is a process to annotate a previously annotated genome by using better bioinformatics methods and more complete databases [9]. Working toward improvement of gene structure as well as functionary information, the importance of genome reannotation has been recognized even before the completion of the first genome sequence [9,10]. However, out of the total number of sequenced microbial genomes (845 at the time of writing), examples of genome-wide reannotations are surprisingly rare [11]. With a few number of documented projects [11-14], nevertheless, several common features can be summarized. Firstly, the functional examination of genes already annotated has become a common practice in reannotation, thanks to the advances of sequence comparison and new experimental data from literature [11-14]. Secondly, new genes may also be described, with evidences mostly from *de novel *gene prediction or sequence comparison to public databases like SWISS-PROT [13], and to a less degree from experimental genome analysis data [12]. Finally, almost all projects involve manual efforts to offer more precise designations to expert curators, and thus help avoid flawed research. In addition to a genome-wide analysis, particular interest may be directed to subsets of genes. For instance, Chen *et al. *[14] focused on assignment of function to genes recognized as being "hypothetical" in previous annotations.

In this work, we combine automated annotation tools with manual efforts to provide a comprehensive and precise reannotation of the *Escherichia coli *CFT073 genome. Hereby we refer to the current release of RefSeq annotation as the original annotation [RefSeq: NC_004431] for CFT073, although the very first annotation in 2002 has already been updated with some minor corrections. With a focus on virulence genes, the reannotation was achieved by using literature curation and applications of several analytical methods including gene finding tools, sequence/domain similarity search and transmembrane region analysis. As a result, 608 coding sequences (CDSs) annotated in RefSeq were excluded, while a total of 299 CDSs are new to the original annotation and one third of these are found in genomic island (GI) regions. Subsequent analysis were conducted by both general and case studies on genes that are crucial during the UTI process, including invasion, colonization, nutrition uptake and population density control. Besides virulence factors, miscellaneous RNAs are believed to contribute to the virulence of strain CFT073 [6]. Therefore, the reannotation presents a total of 40 new miscellaneous RNA genes based on literature curation and database searching. The CFT073 reannotation resource is freely available *via *http://mech.ctb.pku.edu.cn/CFT073/. Following the proposal by Salzberg [10], the reannotation website includes three sections: a brief overview of the methods for reannotation, links to browse the reannotation, and links for data download.

In general, the new CDSs and miscellaneous RNA genes bring new perspectives to the virulence properties of this pathogen. We expect the reannotation to be complementary to the original annotation, with the hope to facilitate the study of new mechanisms of uropathogenicity in CFT073 for a variety of research communities.

## Results & Discussion

### CDS calling and gene start annotation

For the purpose of a systematic reannotation of CDSs, the complete CFT073 sequence has been analyzed upon its all open reading frames (ORFs) longer than 60 bp. All of such ORFs were filtered by running blastp (threshold: e-value < 10^-5 ^and identity > 30%) against Swiss-Prot [15] and rps-blast against the conserved domain database (CDD) [16]. Additional to the blast filter, the results of prediction tools (EasyGene1.2 [17], GeneMark.hmm [18], Glimmer 3.02 [19], and MED 2.0 [20]) were also applied in this study. ORFs co-predicted by at least three of the four tools were included. Comparing to the original annotation, consequently 608 CDSs were removed as listed in Additional File 1. Meanwhile, there was an significant adjustment with totaling 299 CDSs added to the original annotation (see Additional File 2). In fact, all the 608 ruled-out protein-coding genes are either annotated as "hypothetical" or "putative" in the original annotation, and most of them have no function assigned. In contrast, among the 299 new genes, 38 are highly homologous to genes from K-12, or from either one of the two other completely sequenced UPEC strains 536 [RefSeq: NC_008253] and UTI89 [RefSeq: NC_007946]. Moreover, some of the 38's homologues in 536 or UTI89 are related to virulence, such as *cdiAB *[GeneID: 2829859] and *papB *[GeneID: 3992045]. A complete set of the reannotation is included in Additional File 3, meanwhile a comparison between the original annotation and the reannotation is listed in Table 1.

**Table 1:** Overview of the differences between the original RefSeq annotation and the reannotation

	Original annotation (RefSeq: NC_004431)	Reannotation
Genome length	5,231,426 bp	

plasmids	None	

G+C%	50.47%	

Protein-coding genes	5,339	5,030

tRNAs	89	

rRNAs	21	

Miscellaneous RNAs^*a*^	6	46

Backbone genes^*b*^	4,550 (4,440 protein-coding genes, 85 tRNA genes, 21 rRNA genes, and 4 miscellaneous RNA genes)	4,328 (4,178 protein-coding genes, 85 tRNA genes, 21 rRNA genes, and 44 miscellaneous RNA genes)

Genomic island genes^*c*^	905 (899 protein-coding genes, 4 tRNA genes, and 2 miscellaneous RNA genes)	851 (845 protein-coding genes, 4 tRNA genes, and 2 miscellaneous RNA genes)

Cryptic prophages	5	

^*a *^The concept of miscellaneous RNA here includes tRNAs, rRNAs and all other RNAs;

^*b *^In this comparison, we define the genes other than those in the genomic island regions as backbone genes;

^*c *^The genes located in genomic island regions (data conducted by Lloyd and *et al *[47]).

We further manually examined all the pseudogenes (94 samples) in the original annotation. Due to shifting, trimming and splitting, some of the pseudogenes are identified to be protein-coding genes in the reannotation. For example, the annotated pseudogene, c0707 [RefSeq GeneID: 1036199], contains two parts, of which a new gene (c0056r) was reannotated as citrate lyase carrier gene *citD *by gene context analysis. In the reannotation, this new gene is surrounded by citrate carrier protein coding gene *citG *[RefSeq protein_id: NP_752632] and citrate lyase coding genes *citXFE *[RefSeq: NP_752633; NP_752635; NP_752637] in the upstream, and lyase ligase gene *citC *[RefSeq: NP_752638], sensor kinase gene *citA *[RefSeq: NP_752639], and transcriptional regulatory protein coding gene *citB *[RefSeq: NP_752640] in the downstream, and furthermore, is found to be essential to the citrate pathway [21]. Thus the reannotation eliminated the possibility of false interpretation introduced by the original annotation. As a result of the thorough inspection, 35 of the 94 pseudogenes have been directly identified as coding genes newly added into the reannotation, while 55 of them are associated with dozens of new coding genes due to trimming, elongation, splitting or merging along the genomic DNA strand.

Clusters of genes were also manually analyzed in the reannotation. As the most significant characteristic of the *E. coli *CFT073 genome, the GIs, especially PAIs, differ from the backbone genome by possessing clusters of alien genes, especially virulence factor and virulence-associated factor genes. The reannotation indicates that more than one third of the newly added protein-coding genes (102/299 (34.11%)) are located in such genomic regions. Many of these genes are found to be complementary to other genes in genomic islands on both regulation and function levels. For instance, the new microcin genes, *mcmAI *[GeneID: 4194251] and *mchIX *[GeneID: 1039907], from genomic island PAI-CFT073-*serX *are required by the Fur-regulated iron concentration-dependent mirocin secretion (more details will follow).

For TIS annotation, we have proposed a computational method to estimate the annotation accuracy of a sequenced genome [7]. The method calculates the accuracy by estimating the true TIS's contribution to the total sequence pattern around annotated TISs, not by simply comparing one set of predictions to another [7]. As found in that paper, the accuracy of RefSeq TIS-annotation is surprisingly low for CFT073 [7]. This is one of the reasons for us to reannotate this strain. With the increasing number of experimentally verified TISs in other genomes, it will be interesting to take these TIS-already-verified genes as references to improve the annotation of TIS in CFT073. In fact, with an alignment of N-terminal sequences (21 amino acids, 100% identity), this has been implemented as a part of a TIS annotation pipeline previously developed for any genome, namely ProTISA [22]. To have high quality of gene start annotation, herein we applied the ProTISA pipeline for gene start relocation of the CFT073 genome [22].

Briefly, TISs of genes are collected from 1) experimental evidence (including those obtained by alignments of N-terminal sequences; tagged as IPT), 2) conserved domain search (CDC), 3) alignments of orthologous genes (HSC), and 4) predictions from TriTISA [23] for the rest of genes; a complete list can be retrieved from the ProTISA database [22,24]. Although annotated by computational methods, TISs in categories of both CDC and HSC are believed to be highly reliable [22,25,26]. By taking genes with TISs tagged by IPT as benchmarks, the prediction for CFT073 by TriTISA [23] reports an accuracy of 95.6% that is 14.1% higher than that of the RefSeq annotation. In addition, by applying the method proposed in [7], the accuracy of the overall TISs of the reannotation for CFT073 is 19.1% higher than that of the RefSeq annotation (90.0% VS 70.5%). Both are positive towards the high TIS quality of TIS of the reannotation.

### Finding of missed intricacy in PAIs

**Figure 1:**
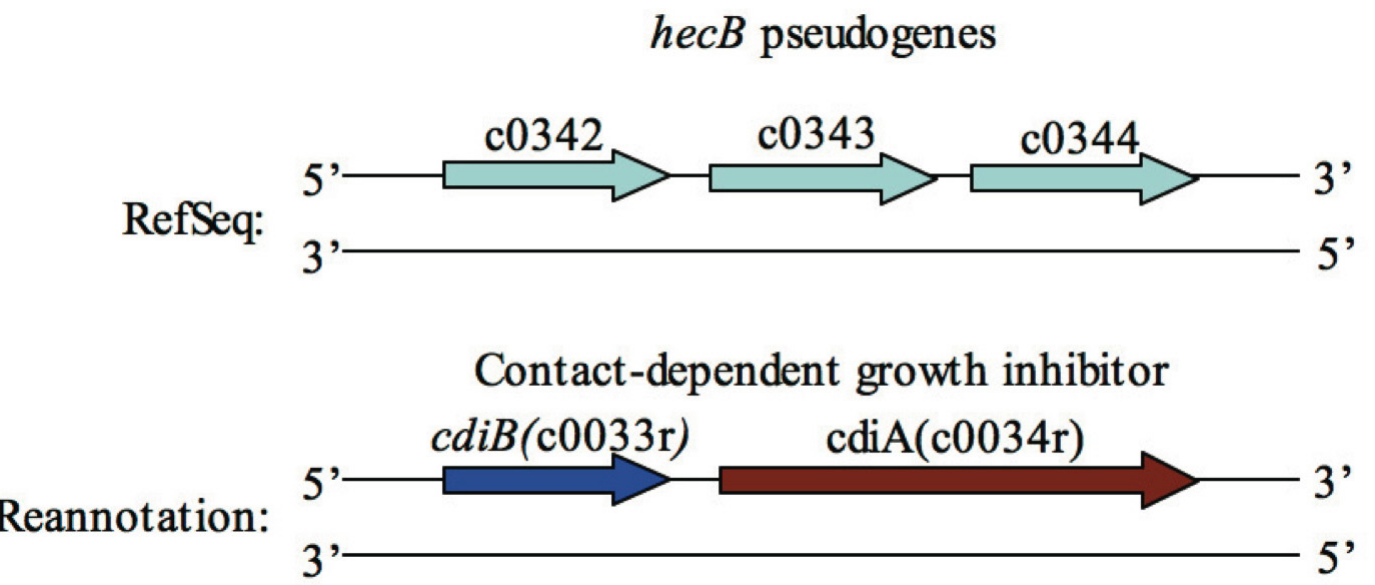
**Differences between RefSeq annotation and the reannotation in cdiAB region**. In the reannotation, three pseudogenes (c0342, c0343 and c0344) are merged into two genes, and are found to be homologues of contact-dependent growth inhibitor encoding genes *cdiAB*.

As mentioned earlier, over one third of the 299 new protein-coding genes (102 genes) in the reannotation are packed in relatively narrow GI regions (796,694 bp in total, about 15% of the whole genome in length). Further analysis demonstrated that 68 of them are located in virulence related genomic island regions, namely pathogenicity islands (PAIs). As the 102 new genes may cooperate with the originally annotated genes to give a more comprehensive PAI scenario, we hereby investigated their functions based on a variety of evidence for use in further studies (see Additional File 4). In this work, case studies are focused on new CDSs in PAI regions, with relevances to other genes from the same region. New genes located in the PAIs offered a more accurate and reasonable interpretation of the virulence regulation in those regions. As listed in Table 2, we found that 12 out of 14 newly designated integrase/transposase genes are located in these regions with potential virulence contribution together with other mobile elements and relevant genes. We applied case study approach in this work for better representations of how the reannotation finds the PAIs' intricacy that was missed in the original annotation. For example, in the pathogenicity island PAI-CFT073-*aspV*, there are 3 consecutive genes (c0342 [GeneID: 1036384], c0343 [GeneID: 1036380], and c0344 [GeneID: 1036375] in RefSeq) originally annotated as pseudogenes. In the reannotation, the three pseudogenes were merged into two new genes (c0033r and c0034r), which are homologues of contact-dependent growth inhibitor genes *cdiA *and *cdiB*, respectively (Figure 1). It is reported that *cdiAB *triggers a non-secretion growth inhibition under certain population density conditions and usually are co-expressed with CdiI, the *cdi *immunity protein, to avoid self-destruction [27]. However, in UPEC strains, *cdiI *is absent from the genome because constitutive P fimbrial expression serves as its substitute [27]. In fact, the P fimbrial operon promoter gene *papB *(c0038r) has been identified located immediately downstream of *cdiAB *in the reannotation, whereas it is missed in the original annotation. Clearly, these three new genes, *cdiA*, *cdiB *and *papB *(as listed in Additional File 4), match up well with the proposed functional relation between P fimbriae and Cdi proteins on the genomic structure level, which are supported by experimental evidences [27].

**Table 2:** List of newly added mobile genetic element-related genes

ID^*a*^	Start site	Stop site	Strand	Comments^*b*^
c0012r	131965	132090	Forward	Transposase for insertion sequence

c0024r	255849	256166	Forward	Transposase protein

c0027r	270919	270674	Reverse	Phage integrase family protein

c0039r	331709	331584	Reverse	Predicted integrase protein

c0042r	376998	377138	Forward	Homologue to Iso-IS1-insB protein

c0053r	627463	627155	Reverse	Putative prophase integrase protein, IntD

c0054r	627782	627483	Reverse	Putative integrase

c0100r	1234499	1235998	Forward	R6-like transposase protein

c0101r	1235995	1236750	Forward	Insertion sequence ATP-binding protein

c0175r	2349098	2349277	Forward	Putative transposase

c0215r	3452165	3451905	Reverse	Insertion sequence protein

c0250r	4284055	4283780	Reverse	IS element

c0251r	4291696	4292046	Forward	Transposase

c0252r	4293121	4293074	Reverse	Transposase IS3/IS911 family protein

^*a *^the ID code follows the order of gene loci on chromosome in the reannotation;

^*b *^the comments on functions are conducted from CDD rps-blast results [16] and Swiss-Prot psi-search results [15].

**Figure 2:**
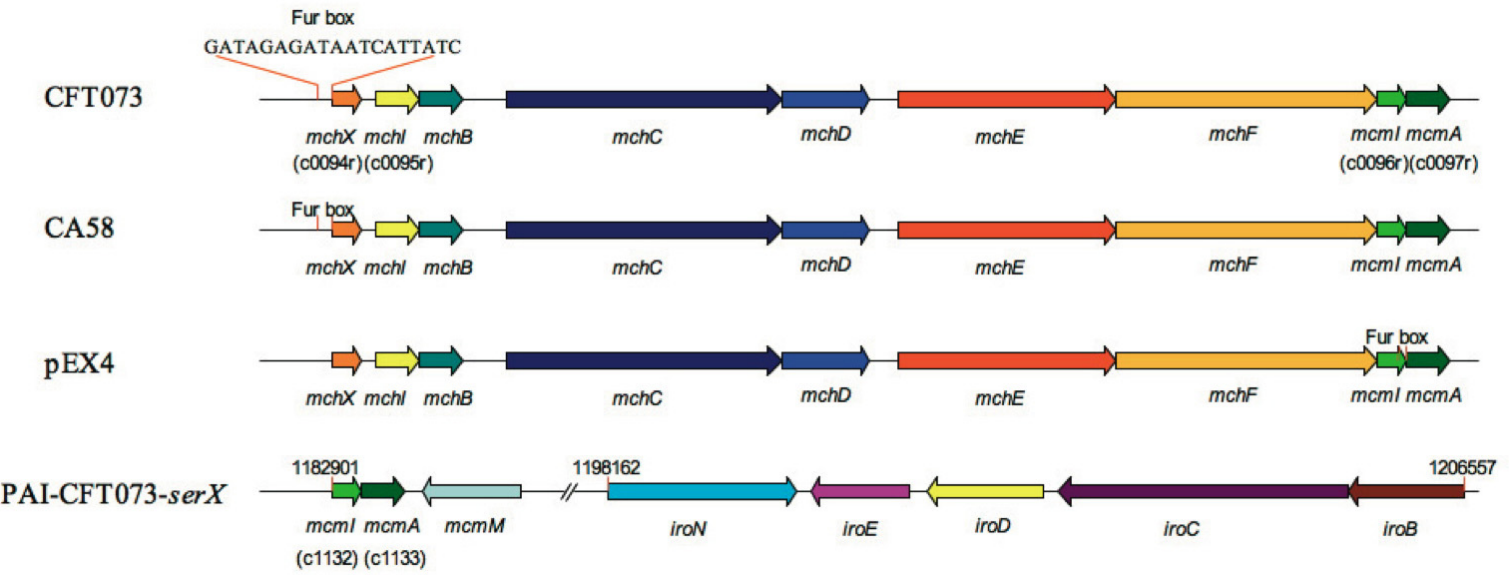
**Conservative structure of mch operon and mcm operon in different E. coli strains**. The Fur boxes are marked by the orange lines; the content in brackets under gene name indicates the ID of this novel gene. The line in the bottom shows the partial structure of PAI-CFT073-*serX*, the numbers on the genes note the positions in the genome.

As another example in the reannotation, the pathogenicity island PAI-CFT073-*serX *contains two new genes (c0097r and c0096r, listed in Additional File 4) that show strong homology to microcin M gene *mcmA *and its immunity protein gene *mcmI*, respectively. Secreted microcins encoded by *mcm *function in population density control under stress, especially during UTI. Interestingly, there are RND-type exporter (microcin M is its transmembrane exportation cargo [28]) system encoding genes, *mchDEF *[RefSeq: NP_753144; NP_753145; NP_753146], located upstream of the two new genes. Such gene arrangements indicate a secreted peptide-based population density control system encoded in PAI-CFT073-*serX *region. Moreover, further upstream another two new genes, *mchX *and *mchI *(c0094r and c0095r, listed in Additional File 4), were recovered in the reannoatation, while a potential Fur-box (conserved sequence for global iron-concentration concerned regulatory protein Fur specific binding) is found upstream of *mchX*. Therefore, the regulation of *mch *genes, and the transportation of *mcmA *and *mcmI*, is potentially controlled by the global iron-concerned factor Fur. Notably, the linear arrangement of *mch *and *mcm *conserved in two other strains CA58 and pEX4, highlights its importance in biological functions (Figure 2). The lack of these genes in the annotation would potentially lead to a mis-interpretation of virulence and evolutionary relations.

### Adjustment in virulence factors

As a uropathogenic strain, CFT073 employs a variety of virulence genes for invasion, adherence, colonization *et al.*, to host cells. Most current studies on this pathogen focus on the virulence factors, such as *fim *operon, antigen 43 and so on, while missing of such genes in the annotation could be misleading for this direction. For example, several critical genes that might contribute to virulence during urinary tract infections, including *hokA *and *hokC *[29] are absent in the original RefSeq annotation. While in the reannotation, dozens of new protein-coding genes show functions relevant to virulence, including 2 toxic membrane genes, 8 cell-wall associated genes, 7 coilcin/microcin genes, 3 fimbrial regulator genes, and 5 outer membrane receptor genes (see Additional File 2). Specially, we list in Additional File 5 a total of 19 new genes that are likely to contribute to the virulence for strain CFT073, of which includes two genes *hokA *and *hokC*. In addition, the reannotation adds a set of small RNA (sRNA) genes which play essential roles in virulence for CFT073 such as *oxyS*, *csrC*, and *omrAB *(see the next subsection).

**Figure 3:**
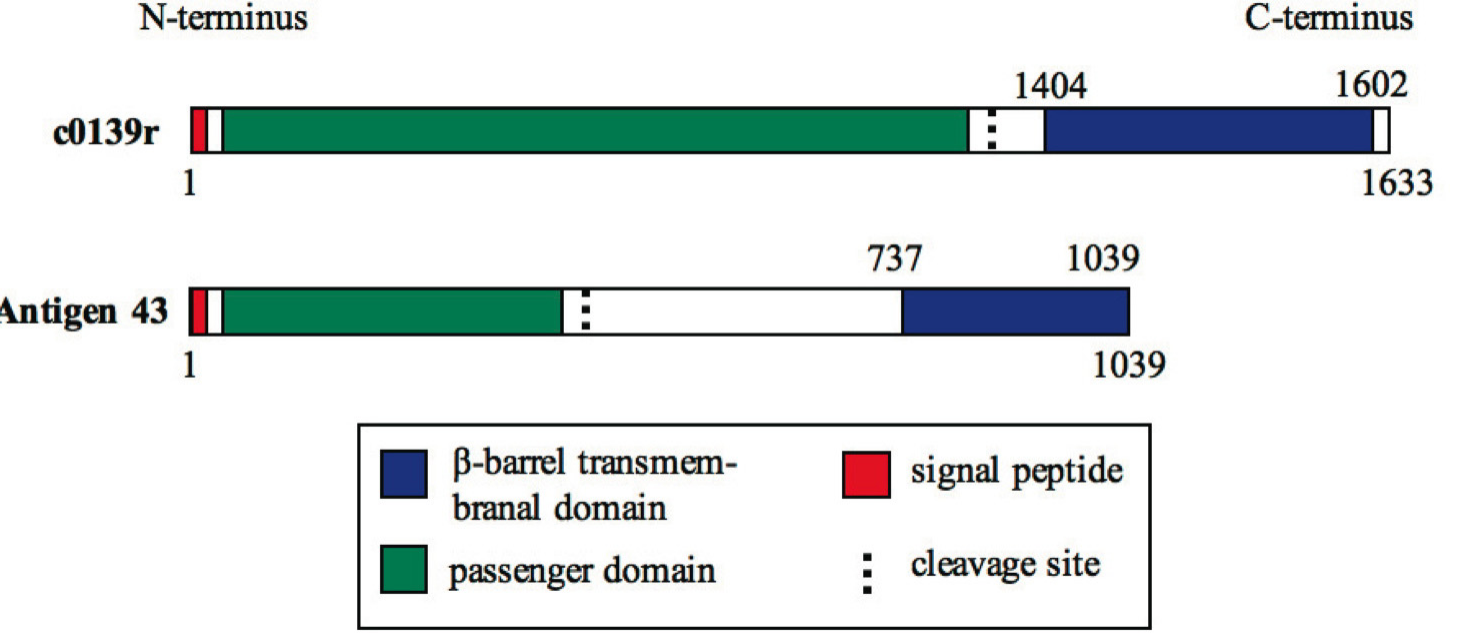
**Structural domains of c0139's product in the reannotation and autotransporter virulence factor antigen 43**. The domain in blue has the ability to form a *β*-barrel, also termed autotransporter domain, and is a key component in self-exportation; the domain in green is the passenger domain, which varies widely with different ATs; the segment in red is signal peptide, which guides the whole protein during the translocation; and the dashed line indicates the cleavage site in which protease cuts AT and releases the passenger segment.

We hereby use case studies to highlight the importance of new coding genes related to virulence. For instance, the newly added CDS c0139r is a candidate for enterobactin siderophore autotransporter (AT). It shows high homology to the EntS/YbdA MFS transporter family members in both UPEC strains UTI89 ([EBI: Q1RC09]; identity: 65%, e-value: 10^-27^) and 536 ([EBI: Q0TI10]; identity: 77%, e-value: 10^-27^). Conserved domain search reveals at least two domains that indicate the AT encoding ability of this new gene c0139r as follows. The C-terminal domains has a strong potential in forming a transmembranal *β*-barrel (HTMSRAP prediction [30]), which is the structural foundation of self-secretion. The passenger domain shows high sequence similarity with haemagluttinin repeat, which is responsible for cell-aggregation through a handshake mechanism [31]. Such structure is conservative among other verified autotransporters such as Antigen 43, a virulence factor mediating cell-cell aggregation that enhances resistance during infections [32] (Figure 3). Thus, the evidence supports that c0139r mediates cell aggregation as a autotransporter encoding gene, and moreover, enhances the diversity of adherence methods of this pathogen and widens its target cell spectrum.

As mentioned earlier, the reannotation discovers the missed *cdiAB *genes and implies a potential regulatory relation between *cdi *genes and P fimbrial expression during population density control. It is interesting that UPEC strains also employ another strategy to mediate their population size and to widen their chance to survive during the infection: secreted antimicrobial peptides such as colicin. The reannotation enriches this category of genes by 7 new genes, including both colicin and immunity proteins (Table 3). For example, the original release annotates ORF c0133 (128997 - 129293) [RefSeq: NP_752086; NC_004431.1] as a hypothetical protein gene. By frame shifting and shortening the pre-annotated ORF, the reannotation determines a colicin gene (c0010r, 127216 - 128997), while the original "hypothetical protein" gene is trimmed into c0011r (129691 - 129984) and assigned as colicin immunity gene. The new gene c0010r gives high sequence similarity with S-type pyocins (identity: 93.3%; e-value: 10^-27^), which is a bacteriocin closely related to colicin E2, E7, and E9. Further conserved domain search for c0010r reveals a congruent linear domain structure with colicins: i) the N-terminal domain has strong homology with the receptor recognizing signal sequence of cytotoxin protein Hcp (pfam06958, COG3157) in *Vibro cholerae *[33], which supports cell surface receptor recognition function of this region; ii) the third domain is homologous to S-type pyocin specific domain, which is responsible for pyocin translocation and penetration; and iii) the C-terminal domain carries the lethal activity and hosts a S-type pyocin specific HNH endonuclease signature. Therefore, the analysis suggests the validation assessment of the function of the new colicin by experiments.

**Table 3:** The newly added colicin and colicin-related genes

ID	Start site	Stop site	Strand	Comments
c0009r	127216	128997	Forward	Uropathogenic specific S-type colicin

c0010r	129401	129688	Forward	Putative colicin

c0011r	129691	129984	Forward	Putative colicin immunity protein

c0094r	1176363	1176527	Forward	Protein MchX

c0095r	1176596	1176805	Forward	Microcin immunity protein, MchI

c0096r	1182901	1183122	Forward	Microcin immunity protein, McmI

c0097r	1183119	1183397	Forward	McmA protein

There are also sets of genes that indirectly contribute to the virulence and are considered virulence factors as well, given that their absence would lead to failure in infections. Particularly, under the extreme environment in the human urinary tract such as high osmotic stress and lack of oxygen, genes in charge of self-adaption are essential to survival in the transition from intestines to its specific niche. Of the new genes in the reannotation, 2 are toxic membrane genes, 8 are cell-wall associated genes, and 5 are outer membrane receptor genes. It is worth noting that some of these are found to be critical in environment sensing and self-adjusting. For instance, c0247r encodes a membrane permeability altering protein which might help CFT073 overcome the high osmolarity in the urinary tract; c0201r produces an anaerobic nitric oxide reductase, which would be essential to CFT073 when in the urinary tract, where oxygen concentration is low and nitrogen is very limited. Therefore, we expect that the reannotation of virulence factors will facilitate a more complete and precise understanding of how this pathogen survives, transfers, and colonizes in human urinary tract.

### Miscellaneous improvements

Mobile genetic elements have been known as being associated with pathogenicity islands in UPEC strains and play an important role in transition from an acute to a chronic state of disease [34,35]. In this regard, the reannotation has also recovered a set of elements such as transposase and integrase. There are in total 14 newly added genes for this category (Table 2). Among them, c0012r is a transposase for insertion sequences, which is located in a prophage repeat region associated with insertion sequence IS629 and another putative transposase gene (c0139 [RefSeq: NP_752091]). Such genomic structure is similar to several other newly designated integrases/transposases. For example, as insertion elements prefer sites around tRNA genes, both c0053r and c0054r are integrase genes next to a *Arg*-tRNA gene, instead of two overlapping pseudogenes as reported in the original annotation. Moreover, the region surrounding c0053r and c0054r is a prophage area and contains phage-related genes such as *nfrAB *[RefSeq: NP_752585; NP_752586] (bacteriophage N4 adsorption genes). However, the picture for these phage-related genes is incomplete in the original annotation because of the missing of transposase/integrase.

To date, about 80 sRNA molecules in *E. coli *have been identified, many of which control transcriptions of virulence-related genes [36]. However, almost all of the essential small RNAs (sRNAs) are found missing in the original RefSeq annotation. To correct this systematic defect, the reannotation carries out an update to sRNA genes. We combined Rfam9.0 prediction [37] and literature investigation for sRNA annotation, and thus retrieved a result of 46 samples (see Additional File 6), in which 6 annotated as miscellaneous genes in RefSeq are also included. Most of these sRNAs' functions are verified by experiments [6]. For instance, *gadY *[GeneID: 2847729] activates a series of reactions in response to the acid environment for better resistance to low pH in the urinary tract, while *ryhB *[GeneID: 2847761] and *fur *[RefSeq: NP_752700] (a global iron-concerned regulator gene) repress each other and thus form a loop to control the expression of iron concentration-dependent genes. With these newly added sRNA genes, it is clear that the reannotation provides a more integral view of the regulatory networks in CFT073.

## Conclusion

Using a combination of approaches and in-depth analysis, the reannotation of the *Escherichia coli *CFT073 genome presents a substantial update across the complete genome. To determine the functional annotation of protein-coding genes and RNAs, we deployed both a series of automated annotation tools and manual efforts, incorporating a wide variety of research information by data integration, literature curation, and genomic comparison against the relative strains in *E. coli*. Major updates include noteworthy correction of all protein-coding genes with 608 from RefSeq annotation being excluded and 299 added, also with 341 where their translation initiation sites were relocated. In addition, 94 pseudogenes annotated in RefSeq were thoroughly inspected and updated. Moreover, the miscellaneous genes (sRNAs) have been updated in number from 6 in the RefSeq to 46 in the reannotation. Based on the adjustment in the reannotation, the concerns are more addressed to new protein-coding genes and sRNAs that are crucial or associated with virulence or the UTI process of CFT073. It is apparent that, without the genes newly added in the reannotation, many important functions or regulatory pathways related to the virulence of strain CFT073 cannot be well illuminated. As a result, the reannotation provides a more comprehensive picture of mechanism of uropathogenicity of this UPEC strain. The new genes change the view of its uropathogenicity in different respects, particularly by new genes in GI regions and new virulence-associated factors. The reannotation can thus serve as an important resource by providing new information of the genomic structure and organization, as well as gene function. We hope that the detailed analysis will facilitate future exploration of novel virulence mechanisms and help guide experimental design.

## Methods

### Sequences

The genome sequences of *E. coli *strains CFT073 [RefSeq: NC_004431] [2], K-12 substrain MG1655 [RefSeq: NC_000913] [38], 536 [Refseq: NC_008253] [39] and UTI89 (with plasmid; [RefSeq: NC_007946] [40] were taken from RefSeq.

### Programs and databases

Predictions of EasyGene1.2 [17] were downloaded from its website. The other three gene-finders, GeneMark.hmm [18], MED 2.0 [20], and Glimmer 3.02 [19], were downloaded, installed and run in local. Other programs include: RPS-blast for conserved domain search (against CDD v2.13 [16]), blastp [41] for similarity search (against SWISS-PROT [13]), gene start prediction with TriTISA [23], sRNA genes prediction based on Rfam9.0 database [37], and ARTEMIS 9 for genome browse [42]. Thresholds of e-value at e10^-5 ^and identity score at 30 are set for blastp and RPS-blast.

### Virulence factor prediction

Multiple sequence alignment with virulence factor sequences from VFDB [43] were manipulated by the uses of EMBOSS suit [44], Mega3.1 [45] and T-coffee [46]. The alignments were automatically shaded according to the default setting of these softwares. The assumed transmembranal protein sequences were examined by HTMSRAP [30].

## Abbreviations

AT: Autotransporter; CDS: Coding sequence; *E. coli*: *Escherichia coli*; GI: Genomic island; ORF: Open reading frame; PAI: Pathogenicity island; TIS: Translation initiation site; UPEC: Uropathogenic *Escherichia coli*; UTI: Urinary tract infection.

## Authors' contributions

CWL carried out the reannotation process. GQH assisted with gene start adjustment, discussed on CDS calling, and make reannotation be publicly accessible to research community. CWL, GQH and HQZ drafted the manuscript. HQZ supervised the study. All authors read and approved the final manuscript.

## Supplementary Material

Additional file 1 **The ruled-out RefSeq genes in the reannotation**. This additional file is a list of the 608 ruled-out RefSeq genes, information includes gene location, strand, gene length, PID, gene synonym code and COG product. Click here for file

Additional file 2 **The newly added CDSs in the reannotation**. This additional file contains the 299 newly added CDSs in the reannotation, information includes gene location, strand, gene length, PID, Gene synonym code and gene product. Click here for file

Additional file 3 **The ptt file for all protein-coding genes**. This additional file encloses all the protein-coding genes in the reannotation, information includes gene ID, position, strand, other database accession number, and function comments. Click here for file

Additional file 4 **The newly added genes in GI regions**. This additional file contains the 102 newly added genes which are located in GI(Genomic Island) regions, information includes PAI ID, position, strand, length, and comments. Click here for file

Additional file 5 **The newly added virulence factor genes**. This additional file contains the 19 newly added genes which contribute to the virulence in pathogen strain CFT073, information includes gene location, strand, gene length, and function comments. Click here for file

Additional file 6 **The miscellaneous RNA genes in the reannotation**. This additional file contains the 46 newly added miscellaneous RNA genes in the reannotation, information includes gene location, strand, gene name, and code in the reannotation. Click here for file
